# Exome Sequencing Reveals Signal Transduction Genes Involved in Impulse Control Disorders in Parkinson's Disease

**DOI:** 10.3389/fneur.2020.00641

**Published:** 2020-07-21

**Authors:** Sabine Prud'hon, Samir Bekadar, Agnès Rastetter, Justine Guégan, Florence Cormier-Dequaire, Lucette Lacomblez, Graziella Mangone, Hana You, Mailys Daniau, Yannick Marie, Hélène Bertrand, Suzanne Lesage, Sophie Tezenas Du Montcel, Mathieu Anheim, Alexis Brice, Fabrice Danjou, Jean-Christophe Corvol

**Affiliations:** ^1^Sorbonne Université, INSERM UMRS 1127, CNRS UMR 7225, Institut du Cerveau et de la Moelle, ICM, Paris, France; ^2^Assistance Publique Hôpitaux de Paris, Centre d'Investigation Clinique neurosciences, Department of Neurology, Hôpital Pitié-Salpêtrière, Paris, France; ^3^Assistance Publique Hôpitaux de Paris, Department of Pharmacology, Hôpital Pitié-Salpêtrière, Paris, France; ^4^Assistance Publique Hôpitaux de Paris, Sorbonne Université, INSERM, Institut Pierre Louis d'Epidémiologie et de Santé Publique, Hôpital Pitié-Salpêtrière, Paris, France; ^5^Hôpitaux Universitaires de Strasbourg, Department of Neurology, Strasbourg, France; ^6^Institut de Génétique et de Biologie Moléculaire et Cellulaire (IGBMC), UMR 7104 CNRS/Unistra, Inserm U1258, Illkirch, France; ^7^Fédération de Médecine Translationnelle de Strasbourg, Université de Strasbourg, Strasbourg, France; ^8^Assistance Publique Hôpitaux de Paris, Department of Genetics, Hôpital Pitié-Salpêtrière, Paris, France

**Keywords:** Parkinson's disease (PD), dopamine agonists (DA), impulse control disorders (ICD), pharmacogenetics, exome sequencing

## Abstract

**Introduction:** Impulse control disorders (ICDs) frequently complicate dopamine agonist (DA) therapy in Parkinson's disease (PD). There is growing evidence of a high heritability for ICDs in the general population and in PD. Variants on genes belonging to the reward pathway have been shown to account for part of this heritability. We aimed to identify new pathways associated with ICDs in PD.

**Methods:** Thirty-six Parkinsonian patients on DA therapy with (*n* = 18) and without ICDs (*n* = 18) matched on age at PD's onset, and gender was selected to represent the most extreme phenotypes of their category. Exome sequencing was performed, and variants with a strong functional impact in brain-expressed genes were selected. Allele frequencies and their distribution in genes and pathways were analyzed with single variant and SKAT-O tests. The 10 most associated variants, genes, and pathways were retained for replication in the Parkinson's progression markers initiative (PPMI) cohort.

**Results:** None of markers tested passed the significance threshold adjusted for multiple comparisons. However, the “Adenylate cyclase activating” pathway, one of the top associated pathways in the discovery data set (*p* = 1.6 × 10^−3^) was replicated in the PPMI cohort and was significantly associated with ICDs in a *post hoc* pooled analysis (combined *p-*value 3.3 × 10^−5^). Two of the 10 most associated variants belonged to genes implicated in cAMP and ERK signaling (rs34193571 in *RasGRF2, p* = 5 × 10^−4^; rs1877652 in *PDE2A, p* = 8 × 10^−4^) although non-significant after Bonferroni correction.

**Conclusion:** Our results suggest that genes implicated in the signaling pathways linked to G protein-coupled receptors participate to genetic susceptibility to ICDs in PD.

## Introduction

Impulse control disorders (ICDs) and related behaviors are defined by the failure to resist an impulse to perform a self-rewarding act that will cause longer-term harm to oneself or others. Their prevalence is estimated between 1 and 3% in the general population while it raised up to 17% in treated Parkinsonian patients (1, 2). Common manifestations in Parkinson's disease (PD) are pathological gambling (PG), hypersexuality, and compulsive eating and buying. Clinical risk factors associated with ICDs in PD include younger age, younger age of PD onset, unmarried status, current smoking, novelty seeking traits, and family history of gambling (1, 3, 4). Above all, ICDs in PD are strongly associated with the dopamine replacement therapy as demonstrated by a similar prevalence of ICDs in untreated Parkinsonian patients as compared to healthy controls (5) and their independent association with dopaminergic drugs, particularly dopamine agonists (1).

A high heritability has been suggested for ICDs in the general population, estimated to 40–60% in twin studies for pathological gambling (6, 7) and in ICDs in PD patients (8). Genetic association studies have found associations with candidate variants in genes coding monoamine and glutamate receptors, transporters, and metabolism enzymes in both the general population (9, 10) and in PD (11–14). ICDs are also more frequent and more severe in PD patients with *PRKN* mutations (15). The reward pathway—involving ventral areas of basal ganglia and its related cortical structures—is thought to play a central role in the pathophysiology of ICDs as suggested in several studies (16, 17). However, these variants explain only a small part of the phenotype's variance, estimated to be 15–21% for pathological gambling in the general population (9).

Rare genetic variants or combinations of variants may explain this and new strategies that are being developed to explore genetic contribution to complex traits. For instance, significant genes in the course of diabetes mellitus or cystic fibrosis have been discovered by comparing exome data of extreme phenotype groups, reasonably supposed to be enriched in causal variants (18, 19). Moreover, when several genetic variants in a gene or group of genes may contribute to the trait, aggregation tests, by evaluating the cumulative effect of multiple variants, are particularly relevant. The power gained with this method, compared to a single test, allows considering a smaller sample (20).

In this proof-of-concept study, we propose an unbiased approach by comparing exome sequences of two extreme phenotypes of Parkinsonian patients with and without ICDs. Our main objective was to identify new genes and pathways associated to ICDs in PD.

## Methods

### Patients and Study Design

Patients were selected from a multicenter, national, case-control study named Behavioral ADdiction and GEnes in Parkinson's disease (BADGE-PD) conducted in France between 2012 and 2015 in 13 centers of the clinical research network for Parkinson's disease (NS-PARK/FCRIN) (21). Three hundred four patients were included with (*n* = 172) or without (*n* = 132) ICDs or related behaviors. Common inclusion criteria were diagnosis of PD according to the UK Parkinson's Disease Society brain bank (22) and age older than 30 years. The study consisted of one visit at the expert center for PD with a neurological and neuropsychological evaluation and a blood sample for DNA extraction. All patients were evaluated by a face-to-face interview with a neuropsychologist with the French PD behavioral scale, validated for the assessment of ICDs and related behaviors in PD (23). This scale explores and quantifies the different components of hyper- and hypo-dopaminergic behaviors in PD, each item being rated on a 5-point scale (severe disorder: 4, marked disorder: 3, moderate disorder: 2, mild disorder: 1, absence of disorder: 0). Patients with ICDs were defined as having two items with a score of ≥ 2 or at least one item with a score of ≥ 2 on the following items: eating behavior, creativity, hobbying, risk-taking behavior, compulsive shopping, punding, pathological gambling, and hypersexuality. Control PD patients were selected to have no more than one of the previous item with a score of 1. All patients had to be of European Caucasian ancestry for at least two generations, and patients with and without ICDs were matched for age and sex. In addition, patients in the control group had more than 5 years since PD diagnosis and were exposed to a dopamine agonist of at least 300 mg of levodopa equivalent daily dose (LEDD). LEDD were calculated as previously described (24). The study was conducted according to international good clinical practice guidelines and submitted to local regulatory and ethical committees. All patients signed an informed consent form prior any procedure. The study was sponsored by the Assistance Publique Hôpitaux de Paris.

For this ancillary study, we selected an extreme phenotype (EP) population corresponding to 36 cases: 18 PD patients with ICDs (cases) and 18 PD patients without ICDs (controls). Cases were selected in descending order of behavioral scale in PD's scores. The five following items were considered: eating behavior, creativity, compulsive shopping, pathological gambling, and hypersexuality. Controls were selected considering the same items if scoring 0 in all of them. Cases and controls were matched for gender and age at PD onset and had been treated with ropinirole or pramipexole, the two major dopamine agonists used in this cohort. When several controls were available for matching, the patient exposed to the highest dose of DA was selected. Finally, as mutations or risk genetic variants have been shown to be associated with neuropsychiatric symptoms in PD patients (15, 25), patients were screened for the G2019S variant on *LRRK2* gene and for pathogenic mutations on *PRKN* gene.

### Replication

For replication, we used the Parkinson's progression markers initiative (PPMI) data set (https://www.michaeljfox.org/ppmi-clinical-study, data downloaded on April 20, 2016), selecting cases and controls with similar criteria than for the hypothesis-generation cohort. In this cohort, ICD behaviors were screened by using the Questionnaire for Impulsive-Compulsive Disorders in Parkinson's disease (QUIP) (26) at each visit. Among the 431 *de novo* Parkinsonian patients included in the cohort, we selected as cases the patients who developed ICD (QUIP>0) during follow-up (*n* = 52) while treating with pramipexole or ropinirole (*n* = 45) with a QUI*P* = 0 at baseline (*n* = 30) and available exome sequencing data (*n* = 27). For controls, we selected patients with QUIP scores remaining null throughout the follow-up (*n* = 127) treated with ropinirole or pramipexole (*n* = 107) with a dose > 290 mg LEDD for more than 3 months (*n* = 29) and exome sequencing data available (*n* = 28). The 10 most associated genetic variants, genes, and pathways were selected for replication using the whole exome sequencing data.

### Sequencing, Variant Calling, and Annotation

Exomes from the 36 selected patients were sequenced on an Illumina NextSeq 500 with the MedExome kit, and the resulting reads were aligned to the hg19 reference genome with BWA applying duplicate removal with Picard Tools MarkedDuplicates. We performed GATK base quality score recalibration, indel realignment, and SNP and INDEL discovery and genotyping across all samples simultaneously using HaplotypeCaller, followed by variant quality score recalibration according to the GATK Best Practices recommendation: GATK-HaplotypeCaller primary QC was applied at the variant level, excluding any variants that were not flagged as “PASS” by the VQSR algorithm, thus excluding variants outside the 95% sensitivity tranches (i.e., the lowest 5% of recalibrated quality scores). Finally, variants were annotated using the SnpEff tool and GENome MINing software (GEMINI, version 0.18.0) (27).

For all 36 samples, mean coverage on target was above 84, and 90% of targeted regions were covered at least at 30X.

### Data Analysis

Genetic variants were filtered to keep variants likely to have a functional impact based on the Combined Annotation Dependent Depletion (CADD) score (28) as made available in GEMINI (27). Expression values of genes were obtained from the Brain eQTL Almanac (http://www.braineac.org). Variants with a CADD score ≥ 12.37 were kept for further analysis, according to published recommendations (29), if also lying in genes expressed in at least one region among putamen, substantia nigra, thalamus, frontal cortex, and temporal cortex. For a pathway to be considered expressed in these brain regions and kept for analysis, 95% or more of its genes should be expressed in at least one of the latter brain areas. Furthermore, pathways were kept for analysis if at least 80% of their genes were represented by at least one variant in the experiment while a minimum of two variants per gene was required for gene-wise test and a minimum allele count of 4 for single-variant testing. For population description, paired sample *t*-test, McNemar, and χ^2^ or Fisher's exact tests were used for group comparisons of quantitative and qualitative variables, using Statistica (version 9.1) software.

Association of single variants with the case/control status was tested using the likelihood ratio test as made available in the Efficient and Parallelizable Association Container Toolbox, version 3.2.6 (EPACTS). Group-wise tests were conducted using SKAT-O tests as made available in EPACTS on both genes and Reactome pathways (30, 31). The significance threshold was adjusted with Bonferroni's method regarding the number of tests ran: 6,953 for variants, 4,769 for genes, and 123 for pathways leading to a threshold for significance of 7.2 × 10^−6^, 1 × 10^−5^, and 4.1 × 10^−4^, respectively.

For the replication stage, combined *p*-values were obtained using the Fisher test when SKAT-O *p-*values were found to be nominally significant in both EP and PPMI cohorts. *P*-values were considered as significant when passing the Bonferroni-corrected significance threshold considering the number of tests run during the discovery and replication phases (6,963 for variants, 4,779 for genes, and 133 for pathways, resulting in a threshold of significance of 7.2 × 10^−6^, 1 × 10^−5^, and 3.8 × 10^−4^). Tests were adjusted on age at PD diagnosis and gender.

## Results

### Population

The demographic and clinical characteristics of the 36 selected patients with extreme phenotypes (cases, *n* = 18; controls, *n* = 18) are described in Table 1. Cases and controls were similar in terms of PD age onset, disease duration, motor severity (MDS-UPDRS 3 and Hoehn & Yahr stage), and cognitive assessment (MMSE). Cases had significantly higher scores than controls at the MDS-UPDRS part I, which was related to a higher score at item I.6 assessing addictive behaviors (respectively, 2.4 vs. 0.8, *p* = 5 × 10^−3^). DA doses were significantly higher in control subjects (370.3 vs. 258.7 mg/day, *p* = 3 × 10^−4^) due to the study design (controls were recruited based on the absence of ICD behaviors despite high doses of DA). Among the cases, all had at least two different ICDs, five patients had a maximum score of 4 for at least one item, and eight patients accumulated at least two types of ICDs with a score equal to 3. There was no difference in ropinirole/pramipexole or gender distributions regarding to the type of ICDs except for hypersexuality, which was exclusively present in males (see Supplementary Data 1).

**Table 1:** Clinical features of the study population.

**Features**	**ICDs + (*n =* 18)**	**ICDs – (*n =* 18)**	***p*-value**
PD age onset, years^m^	49.4 (7.3)	49.8 (7.4)	0.88
Males, n, (%)^m^	11 (61)	11 (61)	1
Disease duration, years^a^	7.5 (4.6)	9.3 (6.2)	0.33
**MDS-UPDRS**			
Part I	2.4 (1.9)	0.8 (1.3)	0.005
Part II	10.4 (3.0)	7.9 (4.2)	0.05
Part III	15.0 (10.3)	18.0 (10.3)	0.38
Part IV	4.5 (3.3)	3 (1.9)	0.13
Hoehn & Yahr stage ≤ 2, n (%)	16 (88.9)	12 (66.7)	0.13
Family history of addiction (alcohol or PG), n (%)	9 (50)	6 (33)	0.25
MMSE	28.8 (1.2)	28.5 (1.7)	0.57
**Treatment**			
Prami/Ropi, n/n (%/%)	9/9 (50/50)	13/5 (72.2/27.8)	0.29
DA LEDD, mg/day	258.7 (96.6)	370.3 (65.9)	0.0003
Total LEDD, mg/day	707.6 (541)	940.3 (379.9)	0.13

*Results are given as mean (SD), otherwise specified*.

m *referred to matching criteria*.

a *at onset of behavioral addictions for cases and at inclusion for control subjects. PG, pathological gambling; MMSE, Mini Mental State Evaluation; Prami, Pramipexole; Ropi, Rominirole. Student t-test for paired samples and the McNemar test were run for quantitative and qualitative variables, respectively*.

### Single-Variant Analysis

After quality controls, 354,174 variants were available for further analyses. Among the 34,981 with a CADD-score ≥ 12.37, 26,418 satisfied the condition of a minimal occurrence of 4 in the cohort for the single test. After selection upon brain expression of genes, 6,953 variants remained for further analyses. The 10 most associated variants are shown in Table 2. Five variants were exonic missense—in *PDCD6IP, RasGRF2, ACAN, COL12A1*, and *OMA1*—and 4 variants were intronic—in *GDA, PGK1, ITGA6*, and *PDE2A*. The last variant was nonsense in the *IL17RB* gene. Two variants (rs34193571 *RasGRF2*, rs1877652 *PDE2A*) were involved in intracellular signal transduction, related to ERK, and cAMP signaling pathways. Three variants were laid on genes belonging to the pathway of focal adhesion (rs2293647, rs3743398, and rs970547 on *ITGA6, ACAN*, and *COL12A1*). No mutation was found in the *LRRK2* or *PRKN* genes.

**Table 2:** Single test association study results (top 10 variants).

**Variant ID**					**Variant distribution in EP cohort**				***p*****-value**	
**ID**	**Gene**	**Annotation**	**MAF (EXAC)**	**MAF (EP)**	**AAC**	**AAC Case**	**AAC Control**	**RR**	**EP**	**PPMI**
rs3203777	PDCD6IP	V383I	0.44	0.50	36	11	25	0.4	0.0001	NS
rs3802506	GDA	intronic	0.16	0.17	12	11	1	11.0	0.0002	*NA*
rs2007039	PGK1	intronic	0.22	0.18	13	0	13	0.0	0.0002	*NA*
rs34193571	RASGRF2	S753P	0.08	0.10	7	7	0	14.0	0.0005	NS
rs2293647	ITGA6	intronic	0.05	0.10	7	7	0	14.0	0.0007	*NA*
rs3743398	ACAN	P864L	0.21	0.22	16	3	13	0.2	0.0008	NS
rs1877652	PDE2A	intronic	0.28	0.31	22	17	5	3.4	0.0008	*NA*
rs970547	COL12A1	G3058S	0.22	0.28	20	4	16	0.3	0.0008	NS
rs1043261	IL17RB	Stop	0.08	0.08	6	0	6	0.0	0.0011	NS
rs17117678	OMA1	I329L	0.10	0.14	10	1	9	0.1	0.0012	NS

*Minor Allele Frequency (MAF) is given according to EXAC data for Caucasian population and in extreme phenotype (EP) cohort. AAC, Alternative Allele Count in all population and in case and control groups; NS, non-significant. RR referred to the ratio of allele frequency in case and in control groups. If the allele count was equal to zero in control group, the value was replaced by 0.5 to enable the division*.

### Genes Analysis

Of the 19,962 genes covered by the MedExome kit, 6,250 were represented by at least two variants with a CADD score ≥ 12.37. After selection upon brain expression, 4,769 genes remained for association testing. The 10 most associated genes upon SKAT-O test are shown in Table 3. Four of them, *DOCK4, RasGRF2, ITGA6*, and *ITGA11*, were linked to the ERK pathway. *PDE2A* belongs to cAMP signaling. For some genes, such as *PDE2A* and *RasGRF2*, association was led by only one frequent variant, which was strongly associated in the single test. For others, as for *DOCK4* and *MYH14*, association resulted from different rare variants laying in this gene in different individuals of the cohort (see Supplementary Data 2).

**Table 3:** SKAT-O on genes results (top 10 genes).

	**EP**		**PPMI**	
**Gene**	**tested variants**	***p-*value**	**tested variants**	***p*-value**
ANAPC5	3 (0)	0.0005	6 (2)	NS
PDE2A	2 (0)	0.0009	10 (2)	NS
FANCA	4 (1)	0.0009	*0*	*NA*
SGK494	2 (0)	0.0013	6 (2)	NS
DOCK4	9 (4)	0.0016	*NA*	*NA*
APOL5	2 (0)	0.0018	4 (1)	NS
RasGRF2	2 (1)	0.0019	*NA*	*NA*
ITGA6	3 (0)	0.0019	18 (1)	NS
ITGA11	7 (4)	0.0020	21 (4)	NS
MYH14	7 (4)	0.0031	26 (3)	NS

*Tested variants are reported as number of tested variants (number of unique variants, i.e., found only once in the cohort)*.

### Pathways Analysis

Of the 1,816 pathways extracted from the Reactome database, 1,130 were represented by at least two variants with a CADD score ≥ 12.37. Among them, only 312 had more than 95% of their genes expressed in the brain's regions of interest, and the 123 that had more than 80% of their genes represented by at least one variant were considered for further analysis. The five most associated pathways using SKAT-O are shown in Table 4. The most associated one (R-HSA-446343, “Localization of the PINCH-ILK-PARVIN complex to focal adhesions” from Reactome database, *p*-value 1.52 × 10^−3^) was related to focal adhesions with a total of eight variants (two of which were unique, i.e., found only once in the cohort) tested. The second most associated pathway was the adenylate activating pathway (R-HSA-170660, “Adenylate cyclase activating pathway”, *p* = 1.56 × 10^−3^) with a total of 29 (10 of which were unique) variants tested distributed on eight genes.

**Table 4:** SKAT-O on pathways results (top 5 pathways).

**Pathway**		**EP**		**PPMI**		**combined *p*-value**
**Name**		**Tested variants**	***p*-value**	**Tested variants**	***p*-value**	
R-HSA-446343	Localization of the PINCH-ILK-PARVIN complex to focal adhesions	8 (2)	0.0015	32 (6)	0.6072	NA
R-HSA-170660	Adenylate cyclase activating pathway	29 (10)	0.0016	29 (10)	0.0210	3.3.10^−5^*^^
R-HSA-5467333	APC truncation mutants are not K63 polyubiquitinated	7 (4)	0.0156	27 (4)	0.925	NA
R-HSA-1483152	Hydrolysis of LPE	5 (4)	0.0231	29 (4)	0.4554	NA
R-HSA-4411364	Binding of TCF/LEF:CTNNB1 to target gene promoters	20 (9)	0.0233	66 (12)	0.4361	NA

*Tested variants are reported as number of tested variants (number of unique variants). EP, Extreme phenotypes cohort. P-value from SKAT-O tests on pathways in EP and in PPMI cohorts are reported. Combined p-values obtained with a Fisher test are reported in the last column. Combined p-values with a Fisher test were only calculated when both p-values in EP and in PPMI cohorts were found nominally significant (i.e., <0.05)*.

* *Significant p-value after Bonferroni correction*.

None of the variants, genes, or pathways tested passed the significance threshold adjusted with Bonferroni's method (significance threshold of 7.2 × 10^−6^, 1 × 10^−5^, and 4.1 × 10^−4^ for a single test, SKAT-O on genes and pathways, respectively), in the discovery phase only.

### Replication

The replication data set consisted of 59 patients (27 cases and 28 controls). Mean age at PD diagnosis was 58 years old in both groups. There were seven women in the cases and seven in the controls. Pramipexole was the most frequent DA in each group: 60 and 76% in cases and controls, respectively. Doses of DA were significantly higher in controls than in cases (372 LEDD vs. 193 mg/day LEDD, *p* < 0.001).

We applied the same analysis for the 10 most associated variants, genes, and pathways. Only the “adenylate cyclase activating pathway” replicated in the PPMI cohort (Table 4, *p* = 2 × 10^−2^ in the PPMI cohort and *p* = 1.56 × 10^−3^ in the EP cohort, resulting in a Fisher combined *p*-value of 3.3 × 10^−5^). Twenty-nine variants were tested in the EP and PPMI cohorts (see Supplementary Data 3 and 4). In the EP cohort, variants laid on eight different genes: *ADCY 1, 3, 4, 5, 6, 8, 9*, and *GNAL*. In the PPMI cohort, the nine genes encoding the adenylate cyclase from 1 to 9 were represented by at least one variant. Six variants of the 29 tested in each cohort were common to both cohorts, resulting in a total of 52 variants tested for this pathway. For five of them (rs3181385 on *ADCY4*, rs3730071, rs115315671, rs55770045 on *ADCY6*, and rs2228949 on *ADCY8*), the effect was observed in the same direction in both cohorts.

## Discussion

In this study comparing whole exome sequences from two extreme phenotypes, we found enrichment of functional variants laying on brain-expressed genes of the “adenylate cyclase activating pathway” in PD patients with ICDs. This result is in accordance with a recent genome-wide association study founding an association between pathological gambling and the cAMP protein kinase signaling (32).

The dopamine replacement therapy that has been strongly associated with ICDs in PD patients activates dopamine receptors, 7-transmembrane domain G protein-coupled receptors (GPCR), regulating adenylate cyclase activity. D2-like receptors inhibit adenylyl cyclase, whereas stimulation of D1-like receptors leads to its activation through Galpha(olf) in the striatum (33). Cyclic-AMP then acts as a second messenger by activating the protein kinase A (PKA), which, in turn, phosphorylates several substrates and activates transcription factors. In the case of concomitant glutamatergic activation, the extracellular regulated kinase (ERK) MAPK pathway is also activated in medium spiny neurons in the striatum (34). These different routes converge to alteration in gene expression (35), which is supposed to underlie long-term neuronal plasticity induced by dopamine in the striatum (36). ERK activation in the ventral striatum has been shown to play a central role in the reward-associated pathway and in addictive behaviors related to alcohol (37) and psychostimulant consumption (38). In mice, activation of the ERK pathway in the dorsal striatum has been shown to underlie abnormal involuntary movements, dyskinesia, developed in response to chronic L-DOPA (39). In our study, the pathway directly related to cAMP signaling the “Adenylate cyclase activating pathway” was found to be associated with ICDs in both EP and PPMI cohorts.

Interestingly, although below the significance threshold, many of the most associated variants or genes tested were linked to the cAMP and ERK-dependent pathways.

The most associated variant in our study was a missense variant (rs3203777) in the *PDCD6IP* gene, also named *DRIP4* for dopamine receptor interacting protein 4. This gene encodes a protein that has been shown to upregulate D1 and D3 receptor expression (40).

In addition, the *rs1877652* variant, enriched in patients with ICDs, is an intronic variant of the *PDE2A* gene encoding the phosphodiesterase 2A controlling cAMP rates and is shown to play a key role in the modulation of the signal induced by dopamine in rodents (41). Seven patients with ICDs and none of the controls had a coding variant on *RasGRF2* (rs34193571), previously associated with alcohol addiction (42), and coding a protein that mediates calcium-dependent activation of the ERK pathway, modulating the presynaptic effect on the activation of the basal ganglia mesolimbic pathway (43).

Several top associated variants, genes, and pathways were related to integrin systems, which also act on ERK signaling via downstream transducers, including the focal adhesion kinase, FAK. Integrin and its downstream effectors have been shown to be involved in structural changes that occurred in the dendritic tree within the addiction to alcohol (44) or cocaine process (45). A rare variant, *rs2293647*, lying in the integrin subunit α 6 gene *ITGA6*, was found only among cases (*p* = 7 × 10^−4^) while the *rs3743398*, a variant lying on the *ACAN* gene belonging to the aggrecan family, was found more frequently in controls, suggesting a protective role. The most associated pathway in the EP cohort was related to the integrin system although it was not replicated in the PPMI cohort.

None of these variants was previously found to be associated with ICDs in PD or in the general population. However, most previous studies focused on candidate gene coding receptors, transporters, or metabolism enzymes of the reward pathway but did not investigate their downstream signaling effectors (9–14).

The main limitation of our study is the small sample sizes of both our hypothesis generation and the replication data sets, which partly resulted from our stringent inclusion criteria (high doses of DA, severe ICDs, or no ICDs at all), and our results deserve further replication in larger cohorts. The underpowered analysis may explain that only one pathway was replicated and that most of the *p*-values obtained were far from reaching the adjustment for the multiple comparison threshold of significance. The lack of replicated results might also be due to differences between the two cohorts in terms of exome sequencing coverage or phenotypic definition of ICDs in the two cohorts. The selection of extreme cases strongly associated with DA-induced ICDs might have biased our results toward genes directly interacting with these drugs, which was the purpose of the design of the study. Our results also suggest that genetic susceptibility to ICDs in PD would rather result from the combination of multigenic variations than unique rare variants with strong effects, which was already suggested for ICDs in the general population (9).

This study highlights the potential important role of enrichment of functional variants in genes coding proteins of intracellular signaling cascades beyond neurotransmitter receptors in the genetic susceptibility of ICDs. Subjects with stronger signal transduction of GPCRs might be at higher risk to develop ICDs when exposed to dopaminergic therapy in PD. How these pathways interfere with drugs or increase the individual susceptibility to ICDs remain to be explored. The combination of clinical, neuroimaging, and exome sequencing data focused on the study of the polymorphism of the different pathways could help in providing some predictability, in the future, for ICDs in PD patients.

## Data Availability Statement

Data are available upon request to any interested researcher or for the purpose of replicating our results (direct requests to jean-christophe.corvol@aphp.fr).

## Ethics Statement

The studies involving human participants were reviewed and approved by CPP Paris VI. The patients/participants provided their written informed consent to participate in this study.

## Author Contributions

J-CC, FD, and SP contributed to the design of the work, data acquisition, interpretation of the data, and draft the manuscript. AR, HB, JG, and YM participated to data analysis. All authors contributed to the interpretation of the data, revised the manuscript for important intellectual content, provide approval for the publication, and agree to be accountable for all aspects of the work.

## Conflict of Interest

J-CC has served on advisory boards for Air Liquide, Biogen, Denali, Ever Pharma, Idorsia, Prevail Therapeutic, Theranexus; and received grants from Ipsen, the Michael J Fox Foundation, and Sanofi. MA received honoraria and travel grants from Johnson and Johnson, Actelion Pharmaceuticals, Teva, LVL, Aguettant, Orkyn, AbbVie. The remaining authors declare that the research was conducted in the absence of any commercial or financial relationships that could be construed as a potential conflict of interest.
