# Supplementary material for: Exome Sequencing Reveals Signal Transduction Genes Involved in Impulse Control Disorders in Parkinson's Disease
Source: Front Neurol. 2020 Jul 21;11:641. doi: 10.3389/fneur.2020.00641 (PMC7385236; doi:10.3389/fneur.2020.00641)
Supplement: Supplementary file 1 [file Table_1.DOCX]

Supplementary data 1; table: Gender and medication distribution regarding type of ICDs

| **ICDs** | **Cases (n=18)** | |  | **p-value** |
| --- | --- | --- | --- | --- |
| compulsive eating | 16 (88.9) | Males/Females | 9/7 (56.3/43.7) | 0.50 |
|  |  | Prami/Ropi | 6/10 (37.5/62.5) | 0.18 |
| hypercreativity | 8 (44.4) | Males/Females | 4/4 (50/50) | 0.63 |
|  |  | Prami/Ropi | 4/4 (50/50) | 1 |
| compulsive buying | 7 (38.9) | Males/Females | 4/3 (57.1/42.9) | 0.77 |
|  |  | Prami/Ropi | 5/2 (71.4/28.6) | 0.14 |
| pathological gambling | 11 (61.1) | Males/Females | 7/4 (63.6/36.4) | 1 |
|  |  | Prami/Ropin | 5/6 (45.5/54.5) | 1 |
| hypersexuality | 7 (38.8) | Males/Females | 7/0 (100/0) | 0.01 |
|  |  | Prami/Ropi | 3/4 (42.9/57.1) | 1 |

Legend: results are given as number of patients (percentage). Prami= Pramipexole. Ropi= Ropinirole Proportion were compared using Fisher’s exact test.
