# Supplementary material for: Exome Sequencing Reveals Signal Transduction Genes Involved in Impulse Control Disorders in Parkinson's Disease
Source: Front Neurol. 2020 Jul 21;11:641. doi: 10.3389/fneur.2020.00641 (PMC7385236; doi:10.3389/fneur.2020.00641)
Supplement: Supplementary file 2 [file Table_2.DOCX]

Supplementary data 2; table: variants ‘characteristics of the ten most associated genes

| **Variant’s characteristics** | | | | | **Variant’s distribution in EP cohort** | | | | | **p-value** |
| --- | --- | --- | --- | --- | --- | --- | --- | --- | --- | --- |
| Gene | Chr:pos_ref/alt | ID | Annotation | MAF | MAF (EP) | AAC total | AAC case | AAC control | RR | EP cohort |
| *ANAPC5* | 12:121756058_A/G | rs118176996 | intronic | 0.01 | 0.03 | 2 | 2 | 0 | 4.0 | *NA* |
|  | 12:121768248_T/C | rs2280067 | intronic | 0.09 | 0.11 | 8 | 6 | 2 | 3.0 | 0.1025 |
|  | 12:121769265_T/C | rs62620751 | intronic | 0.05 | 0.11 | 8 | 6 | 2 | 3.0 | 0.1025 |
| *PDE2A* | 11:72291927_T/C | rs577536 | splicing | 0.99 | 1.00 | 72 | 36 | 36 | 1.0 | *NA* |
|  | 11:72307742_G/A | rs1877652 | intronic | 0.27 | 0.31 | 22 | 17 | 5 | 3.4 | 0.0008 |
| *FANCA* | 16:89815152_G/A | rs17233497 | missense | 0.07 | 0.10 | 7 | 6 | 1 | 6.0 | 0.0252 |
|  | 16:89842176_C/G | rs139235751 | missense | 0.01 | 0.01 | 1 | 1 | 0 | 2.0 | *NA* |
|  | 16:89857935_G/A | rs11646374 | missense | 0.06 | 0.10 | 7 | 6 | 1 | 6.0 | 0.0252 |
|  | 16:89883007_A/T | rs1800282 | missense | 0.09 | 0.04 | 3 | 3 | 0 | 6.0 | *NA* |
| *SGK494* | 17:26938651_C/T | rs34026109 | missense | 0.03 | 0.04 | 3 | 3 | 0 | 6.0 | *NA* |
|  | 17:26939612_T/A | rs117006142 | intronic | 0.02 | 0.04 | 3 | 3 | 0 | 6.0 | *NA* |
| *DOCK4* | 7:111368481_G/A | rs199706346 | missense | 0.01 | 0.01 | 1 | 1 | 0 | 2.0 | *NA* |
|  | 7:111368491_C/T | rs12705795 | missense | 0.07 | 0.06 | 4 | 4 | 0 | 8.0 | 0.0111 |
|  | 7:111372244_G/T | rs12705796 | intronic | 0.32 | 0.25 | 18 | 13 | 5 | 2.6 | 0.0217 |
|  | 7:111379198_G/C | rs150569245 | missense | 0.01 | 0.01 | 1 | 1 | 0 | 2.0 | *NA* |
|  | 7:111386365_G/C | rs3735535 | intronic | 0.03 | 0.35 | 25 | 11 | 14 | 0.8 | 0.4115 |
|  | 7:111487280_A/G |  | intronic |  | 0.01 | 1 | 1 | 0 | 2.0 | *NA* |
|  | 7:111512032_A/T | rs10224326 | intronic | 0.20 | 0.21 | 15 | 9 | 6 | 1.5 | 0.3636 |
|  | 7:111517227_T/C | rs12705801 | missense | 0.01 | 0.01 | 1 | 0 | 1 | 0.0 | *NA* |
|  | 7:111580166_T/C | rs144867634 | missense | 0.02 | 0.03 | 2 | 2 | 0 | 4.0 | *NA* |
| *APOL5* | 22:36122930_C/T | rs2076671 | missense | 0.29 | 0.29 | 21 | 12 | 9 | 1.3 | 0.3961 |
|  | 22:36123083_C/T | rs2076672 | missense | 0.25 | 0.28 | 20 | 15 | 5 | 3.0 | 0.0033 |
| *RasGRF2* | 5:80409526_T/C | rs34193571 | missense | 0.07 | 0.10 | 7 | 7 | 0 | 14.0 | 0.0005 |
|  | 5:80419590_A/G | rs112269950 | splicing | 0.01 | 0.01 | 1 | 1 | 0 | 2.0 | *NA* |
| *ITGA6* | 2:173292379_G/A |  | Utr5 |  | 0.08 | 6 | 6 | 0 | 12.0 | 0.0020 |
|  | 2:173339808_G/A | rs11895564 | missense | 0.28 | 0.33 | 24 | 13 | 11 | 1.2 | 0.5622 |
|  | 2:173344680_A/C | rs2293647 | intronic | 0.04 | 0.10 | 7 | 7 | 0 | 14.0 | 0.0007 |
| *ITGA11* | 15:68605133_G/A | rs201932761 | missense | <0.001 | 0.01 | 1 | 1 | 0 | 2.0 | *NA* |
|  | 15:68605169_G/A | rs4777035 | missense | 0.59 | 0.43 | 31 | 15 | 16 | 0.9 | 0.8127 |
|  | 15:68612714_C/T | rs201394529 | missense | <0.001 | 0.01 | 1 | 1 | 0 | 2.0 | *NA* |
|  | 15:68628163_C/T | rs2306022 | missense | 0.07 | 0.04 | 3 | 3 | 0 | 6.0 | *NA* |
|  | 15:68631740_A/G |  | intronic |  | 0.10 | 7 | 5 | 2 | 2.5 | 0.1926 |
|  | 15:68631894_C/T | rs61729767 | missense | 0.01 | 0.01 | 1 | 1 | 0 | 2.0 | *NA* |
|  | 15:68653946_C/T | rs148886354 | missense | <0.01 | 0.01 | 1 | 1 | 0 | 2.0 | *NA* |
| *MYH14* | 19:50713713_C/A | rs590722 | missense | 0.14 | 0.10 | 7 | 3 | 4 | 0.8 | 0.6679 |
|  | 19:50720949_G/A | rs34773557 | missense | 0.01 | 0.01 | 1 | 0 | 1 | 0.0 | *NA* |
|  | 19:50728955_G/A | rs12983888 | intronic | 0.32 | 0.32 | 23 | 10 | 13 | 0.8 | 0.4239 |
|  | 19:50770338_T/G |  | intronic |  | 0.01 | 1 | 0 | 1 | 0.0 | *NA* |
|  | 19:50792684_C/T | rs45591233 | intronic | 0.04 | 0.03 | 2 | 0 | 2 | 0.0 | *NA* |
|  | 19:50792770_G/C |  | missense |  | 0.01 | 1 | 0 | 1 | 0.0 | *NA* |
|  | 19:50810311_G/A | rs371404005 | missense | <0.001 | 0.01 | 1 | 0 | 1 | 0.0 | *NA* |

Legend: same nomenclature as in table 2 is applied. Chromosome position is reported according to hg19 version.
