# Supplementary material for: Exome Sequencing Reveals Signal Transduction Genes Involved in Impulse Control Disorders in Parkinson's Disease
Source: Front Neurol. 2020 Jul 21;11:641. doi: 10.3389/fneur.2020.00641 (PMC7385236; doi:10.3389/fneur.2020.00641)
Supplement: Supplementary file 3 [file Table_3.DOCX]

Supplementary data 3; table: variants ‘characteristics tested in the adenylate cyclase activating pathway in the extreme phenotypes cohort

| **Variant’s characteristics** | | | | | **Variant’s distribution in EP cohort** | | | | | **EP cohort single variant testing** |
| --- | --- | --- | --- | --- | --- | --- | --- | --- | --- | --- |
| **Gene** | **Chr:pos_ref/alt** | **ID** | **Annotation** | **MAF** | **MAF (EP)** | **AAC total** | **AAC case** | **AAC control** | **RR** | **p-value** |
| *ADCY1* | 7:45632537_G/A | rs760384440 | intronic | < 0.001 | 0.01 | 1 | 1 | 0 | 2 | NA |
|  | 7:45753597_G/T | rs12721481 | Utr3 | <0.001 | 0.01 | 1 | 1 | 0 | 2 | NA |
|  | 5:7520881_G/T | rs13166360 | missense | 0.18 | 0.31 | 22 | 10 | 12 | 0.8 | 0.5715 |
| *ADCY3* | 2:25047327_G/A | rs199524620 | missense | 0.00 | 0.01 | 1 | 1 | 0 | 2 | NA |
| *ADCY4* | 14:24787588_T/C | rs3181385 | Utr3 | 0.06 | 0.10 | 7 | 4 | 3 | 1.3 | 0.673 |
|  | 14:24792132_A/G | rs74387312 | missense | 0.00 | 0.01 | 1 | 1 | 0 | 2 | NA |
|  | 14:24794905_C/T | rs17102928 | intronic | 0.11 | 0.04 | 3 | 2 | 1 | 2 | NA |
|  | 14:24799578_C/G | rs12436417 | intronic | 0.29 | 0.28 | 20 | 9 | 11 | 0.8 | 0.6242 |
|  | 14:24799586_G/A | rs74896270 | intronic | 0.06 | 0.03 | 2 | 1 | 1 | 1 | NA |
|  | 14:24800879_A/G | rs3181256 | intronic | 0.31 | 0.29 | 21 | 9 | 12 | 0.8 | 0.4566 |
| *ADCY5* | 3:123036962_G/A |  | synonym. | NA | 0.01 | 1 | 1 | 0 | 2 | NA |
|  | 3:123039584_C/T | rs112050992 | intronic | 0.02 | 0.01 | 1 | 1 | 0 | 2 | NA |
|  | 3:123049938_A/C | rs6806529 | intronic | (A) 0.31 | 0.46 | 33 | 16 | 17 | 0.9 | 0.8259 |
|  | 3:123167338_C/T | rs550254165 | missense | < 0.001 | 0.01 | 1 | 1 | 0 | 2 | NA |
| *ADCY6* | 12:49162317_C/G |  | Utr3 | NA | 0.01 | 1 | 1 | 0 | 2 | NA |
|  | 12:49167202_A/C | rs3729980 | intronic | 0.15 | 0.18 | 13 | 4 | 9 | 0.4 | 0.0703 |
|  | 12:49167683_C/A | rs3730074 | intronic | 0.12 | 0.18 | 13 | 4 | 9 | 0.4 | 0.0703 |
|  | 12:49168798_C/A | rs3730071 | missense | 0.04 | 0.03 | 2 | 1 | 1 | 1 | NA |
|  | 12:49168848_G/C | rs3730070 | intronic | 0.17 | 0.18 | 13 | 4 | 9 | 0.4 | 0.0703 |
|  | 12:49176805_C/T | rs115315671 | missense | 0.03 | 0.03 | 2 | 2 | 0 | 4 | NA |
|  | 12:49176860_G/A | rs55770045 | missense | 0.02 | 0.03 | 2 | 2 | 0 | 4 | NA |
| *ADCY8* | 8:131897082_G/A | rs4128982 | intronic | 0.25 | 0.32 | 23 | 11 | 12 | 0.9 | 0.7981 |
|  | 8:131916318_G/A | rs11781997 | intronic | (G) 0.44 | 0.47 | 34 | 15 | 19 | 0.8 | 0.3422 |
|  | 8:132002814_C/T | rs11991124 | intronic | 0.22 | 0.24 | 17 | 12 | 5 | 2.4 | 0.0614 |
|  | 8:132052342_C/T | rs2228949 | missense | 0.09 | 0.01 | 1 | 1 | 0 | 2 | NA |
| *ADCY9* | 16:4057603_G/A | rs3730119 | intronic | 0.20 | 0.21 | 15 | 7 | 8 | 0.9 | 0.7594 |
| *GNAL* | 18:11752943_C/A | rs1895689 | intronic | 0.30 | 0.28 | 20 | 7 | 13 | 0.5 | 0.0889 |
|  | 18:11754034_T/C | rs72865259 | intronic | 0.16 | 0.15 | 11 | 4 | 7 | 0.6 | 0.2735 |
|  | 18:11873994_G/T | rs117377308 | intronic | < 0.01 | 0.01 | 1 | 1 | 0 | 2 | NA |

Legend: same nomenclature as in table 2 is applied. Chromosome position is reported according to hg19 version.
