# Supplementary material for: Exome Sequencing Reveals Signal Transduction Genes Involved in Impulse Control Disorders in Parkinson's Disease
Source: Front Neurol. 2020 Jul 21;11:641. doi: 10.3389/fneur.2020.00641 (PMC7385236; doi:10.3389/fneur.2020.00641)
Supplement: Supplementary file 4 [file Table_4.DOCX]

Supplementary data 4; table: variants ‘characteristics tested in the adenylate cyclase activating pathway in the PPMI cohort

| **Variant’s characteristics** | | | | | **Variant’s distribution in PPMI cohort** | | | | | **PPMI cohort single variant testing** |
| --- | --- | --- | --- | --- | --- | --- | --- | --- | --- | --- |
| **Gene** | **Chr:pos_ref/alt** | **ID** | **Annotation** | **MAF** | **MAF (PPMI)** | **AAC total** | **AAC case** | **AAC control** | **RR** | **p-value** |
| *ADCY1* | 7:45632443_C/T | rs75000917 | missense | <0.001 | 0.01 | 1 | 0 | 1 | 0.0 | NA |
|  | 7:45753597_G/T | rs12721481 | Utr3 | <0.001 | 0.01 | 1 | 0 | 1 | 0.0 | NA |
|  | 7:45756894_C/A |  | Utr3 |  | 0.04 | 4 | 1 | 3 | 0.3 | 0.3060 |
|  | 7:45756997_C/G |  | Utr3 |  | 0.22 | 24 | 10 | 14 | 0.7 | 0.3317 |
|  | 7:45761351_G/A |  | Utr3 |  | 0.09 | 10 | 7 | 3 | 2.3 | 0.1394 |
|  | 7:45762696_G/A |  | Utr3 |  | 0.20 | 22 | 11 | 11 | 1.0 | 0.9232 |
| *ADCY2* | 5:7520881_G/T | rs13166360 | missense | 0.25 | 0.28 | 31 | 14 | 17 | 0.8 | 0.5990 |
|  | 5:7707862_G/A | rs140842335 | missense | <0.001 | 0.01 | 1 | 0 | 1 | 0.0 | NA |
|  | 5:7766894_G/A | rs184691479 | missense | <0.001 | 0.01 | 1 | 0 | 1 | 0.0 | NA |
| *ADCY3* | 2:25042211_T/G |  | Utr3 |  | 0.01 | 1 | 0 | 1 | 0.0 | NA |
|  | 2:25061466_T/C |  | missense |  | 0.01 | 1 | 0 | 1 | 0.0 | NA |
|  | 2:25141546_T/C | rs143313188 | missense | <0.001 | 0.01 | 1 | 0 | 1 | 0.0 | NA |
| *ADCY4* | 14:24787588_T/C | rs3181385 | Utr3 | 0.06 | 0.10 | 11 | 6 | 5 | 1.2 | 0.6857 |
|  | 14:24787600_T/A |  | Utr3 |  | 0.01 | 1 | 1 | 0 | 2.0 | NA |
|  | 14:24788316_G/A | rs61741640 | missense | <0.002 | 0.01 | 1 | 1 | 0 | 2.0 | NA |
|  | 14:24791274_C/T |  | missense |  | 0.01 | 1 | 0 | 1 | 0.0 | NA |
|  | 14:24803710_G/A | rs77202343 | missense | 0.05 | 0.02 | 2 | 1 | 1 | 1.0 | NA |
| *ADCY5* | 3:123046482_G/A |  | missense | <0.001 | 0.01 | 1 | 1 | 0 | 2.0 | NA |
| *ADCY6* | 12:49161396_G/T |  | Utr3 |  | 0.02 | 2 | 2 | 0 | 4.0 | NA |
|  | 12:49165650_C/T | rs143958339 | missense | <0.002 | 0.01 | 1 | 1 | 0 | 2.0 | NA |
|  | 12:49168798_C/A | rs3730071 | missense | 0.04 | 0.02 | 2 | 1 | 1 | 1.0 | NA |
|  | 12:49176805_C/T | rs115315671 | missense | 0.03 | 0.08 | 9 | 7 | 2 | 3.5 | 0.0541 |
|  | 12:49176860_G/A | rs55770045 | missense | 0.02 | 0.01 | 1 | 1 | 0 | 2.0 | NA |
| *ADCY7* | 16:50325653_T/A | rs138162056 | missense | <0.001 | 0.01 | 1 | 0 | 1 | 0.0 | NA |
|  | 16:50338341_C/T | rs61731915 | missense | 0.01 | 0.02 | 2 | 0 | 2 | 0.0 | NA |
| *ADCY8* | 8:132051970_C/A | rs75246765 | missense | 0.02 | 0.01 | 1 | 0 | 1 | 0.0 | NA |
|  | 8:132052342_C/T | rs2228949 | missense | 0.09 | 0.05 | 5 | 5 | 0 | 10.0 | NA |
| *ADCY9* | 16:4163754_T/G | rs52791170 | missense | 0.01 | 0.02 | 2 | 0 | 2 | 0.0 | NA |
|  | 16:4165817_C/T |  | Utr5 |  | 0.03 | 3 | 0 | 3 | 0.0 | NA |

Legend: same nomenclature as in table 2 is applied. Chromosome position is reported according to hg19 version.
